# Supplementary material for: Metabolome and Microbiome Analysis to Study the Flavor of Summer Black Tea Improved by Stuck Fermentation
Source: Foods. 2023 Sep 13;12(18):3414. doi: 10.3390/foods12183414 (PMC10527649; doi:10.3390/foods12183414)
Supplement: Supplementary file 1 [file foods-12-03414-s001.zip › Supplementary data (Figures S1-S6).pdf]

Figure S1. Diagram of tea preparation.

Figure S2. Sample quality control analysis. (A-B) Total ions current (TIC) overlapping map of mass spectrometry data from quality control samples (QC) samples. (C-D) A multi-peak detection plot of the metabolites. (E) Coefficient of variation of QC. The horizontal coordinate is the retention time of the metabolite detection and the vertical coordinate is the ion flow intensity of the ion detection (The intensity units are counts per second (cps)). (A, C) in positive ion mode, (B, D) in negative ion mode.

Figure S3. Permutation tests of the OPLS-DA models in LC-MS/MS. The permutation tests were carried out with 200 random permutations.

Figure S4. Sample quality control analysis. (A) TIC of quality control samples (QC). (B) Overlay of TIC for mass spectrometric detection of QC samples. (C) Coefficient of variation of QC, YH and FHYH. The horizontal coordinate is the retention time of the metabolite detection and the vertical coordinate is the ion flow intensity of the ion detection (The intensity units are counts per second (cps)).

Figure S5. Permutation tests of the OPLS-DA models in GC-MS/MS. The permutation tests were carried out with 200 random permutations.

Figure S6. Alpha diversity of microbial community. (A) Shannon index of fungi. (B) Simpson index of fungi. (C) Shannon index of bacteria. (D) Simpson index of bacteria.

Figure S1.

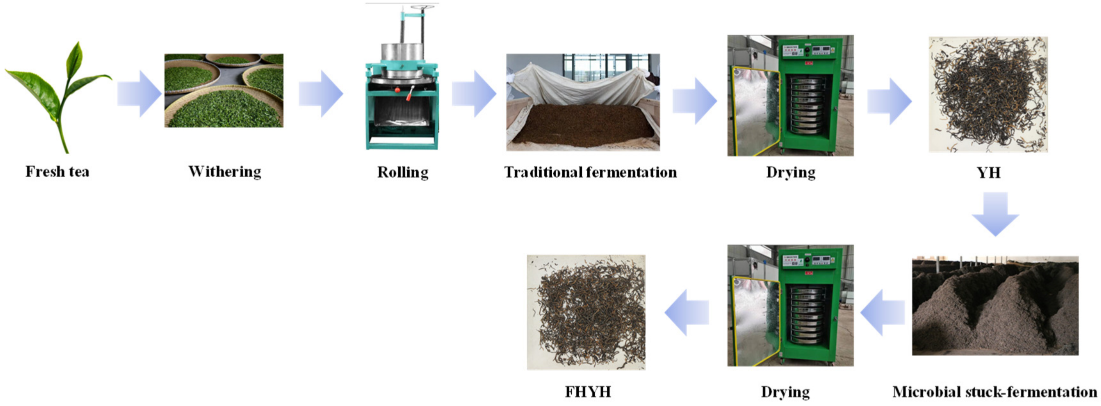

Figure S2.

A

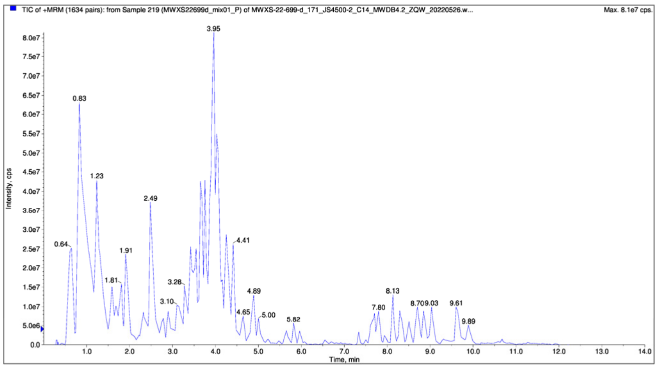

B

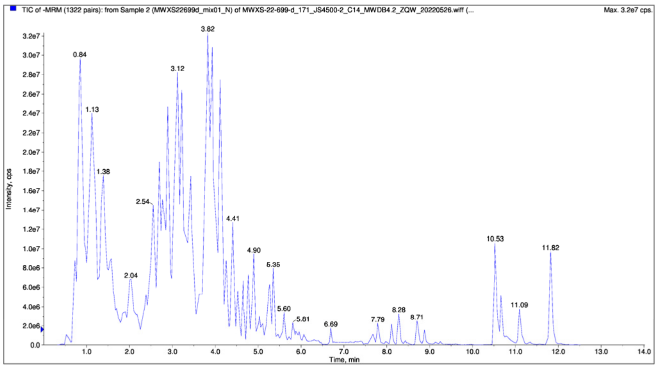

C

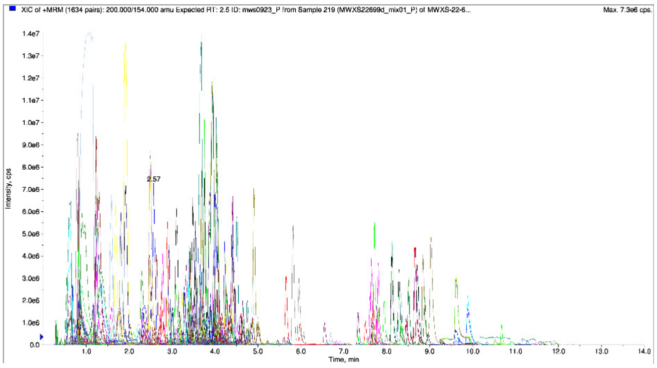
XIC of +MRM (1634 pairs): 200.000/154.000 amu Expected RT: 2.5 ID: mws0923_P from Sample 219 (MWXS22699d_mix01_P) of MWXS-22-6...
Max. 7.3e6 cps.
Intensity, cps
Time, min
2.57

D

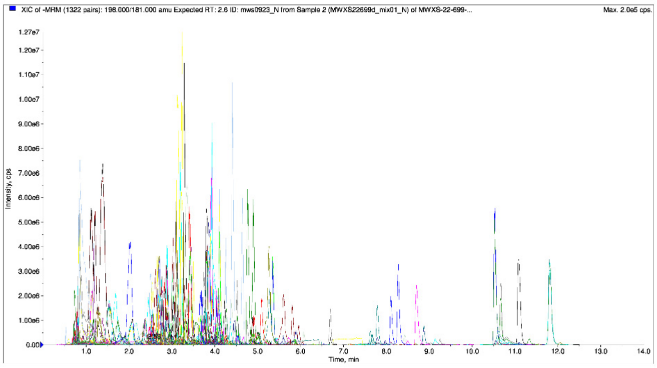
XIC of -MRM (1322 pairs): 198.000/181.000 amu Expected RT: 2.6 ID: mws0923_N from Sample 2 (MWXS22699d_mix01_N) of MWXS-22-699-...
Max. 2.0e5 cps.
Intensity, cps
Time, min

E

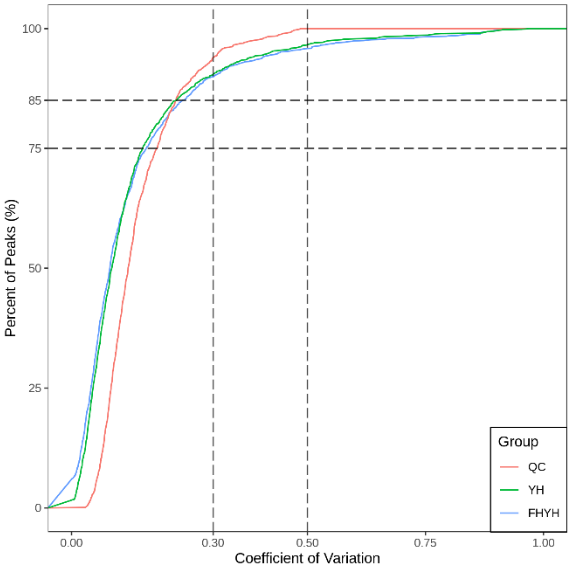
Percent of Peaks (%)
Coefficient of Variation
Group
QC
YH
FHYH

Figure S3

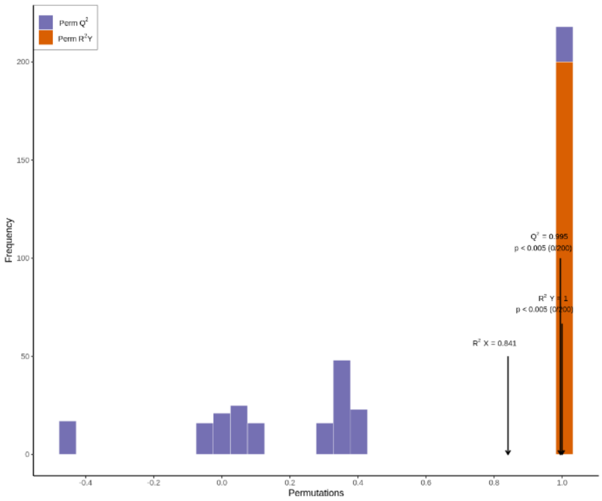

Figure S4

A

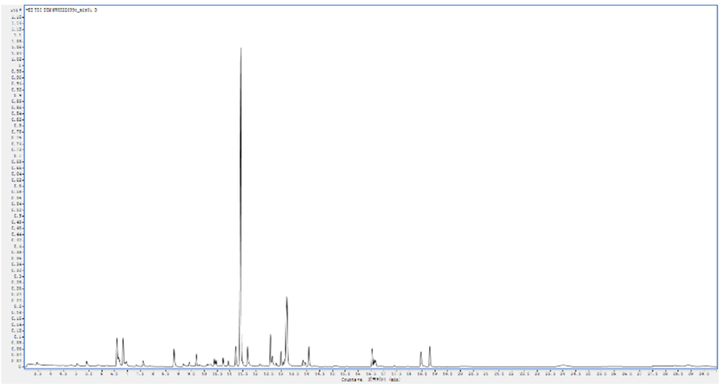

B

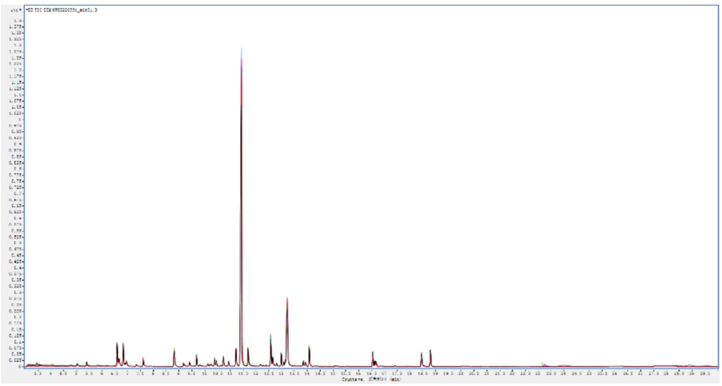

C

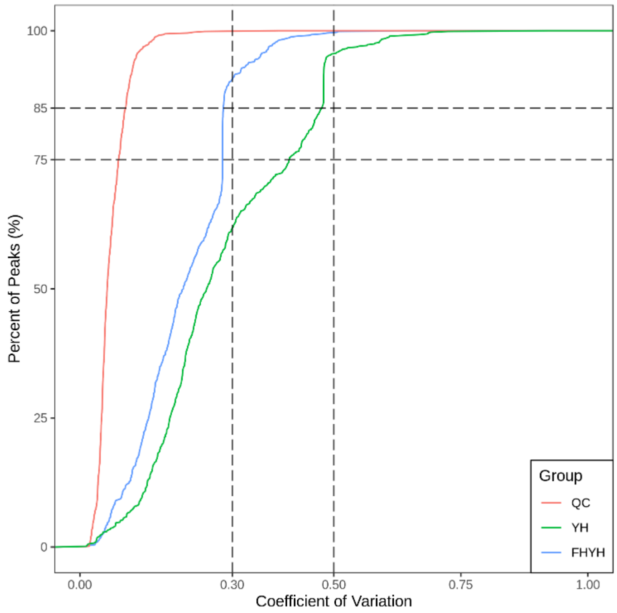

Figure S5

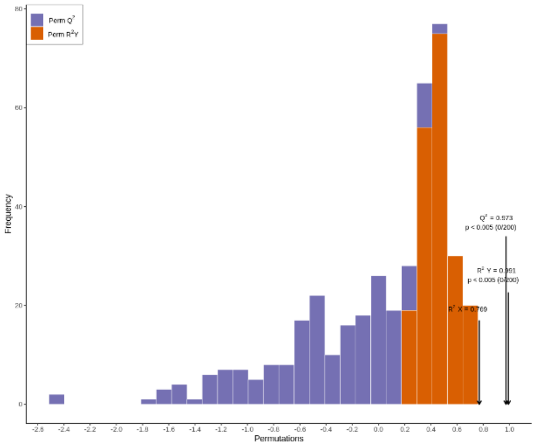

Figure S6

A

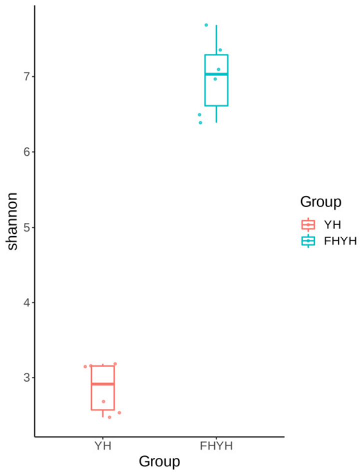

B

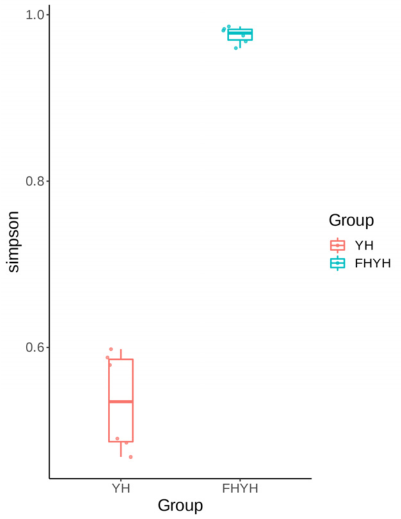
1.0
0.8
0.6
simpson
YH
FHYH
Group
Group
YH
FHYH

C

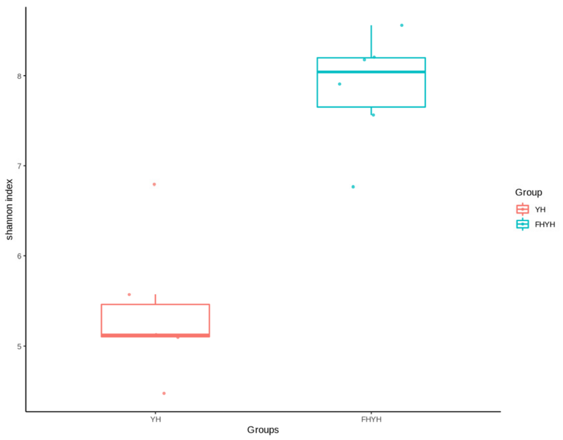
8
7
6
5
shannon index
YH
FHYH
Groups
Group
YH
FHYH

D

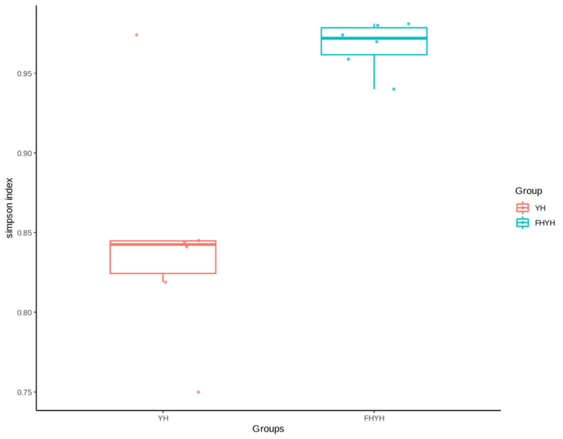
0.95
0.90
0.85
0.80
0.75
simpson index
YH
FHYH
Groups
Group
YH
FHYH
